# Supplementary material for: Chronic Stress That Changed Intestinal Permeability and Induced Inflammation Was Restored by Estrogen
Source: Int J Mol Sci. 2023 Aug 15;24(16):12822. doi: 10.3390/ijms241612822 (PMC10454097; doi:10.3390/ijms241612822)
Supplement: Supplementary file 1 [file ijms-24-12822-s001.zip › ijms-2511935-supplementary.pdf]

Supplementary figure

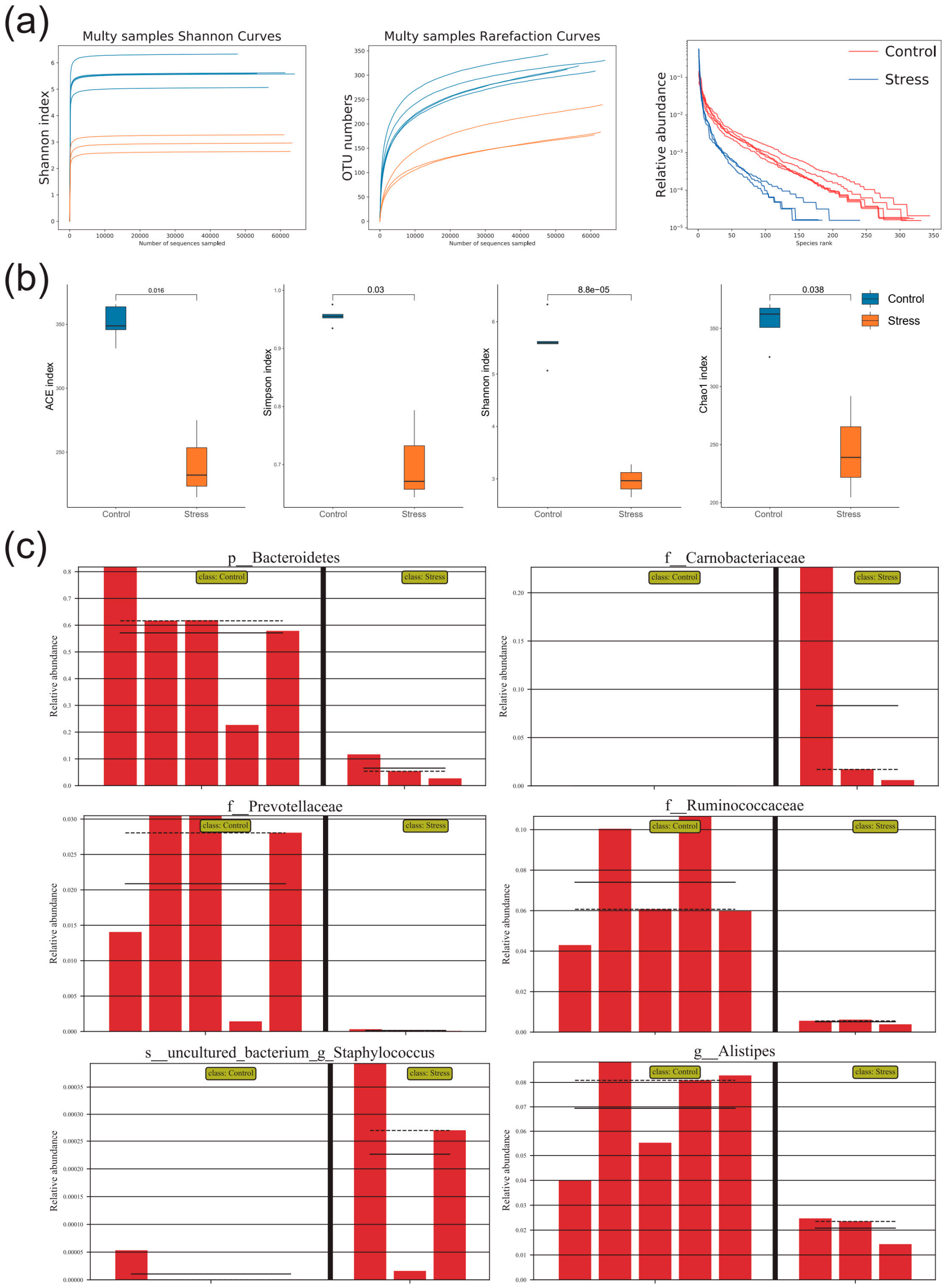

Supplement Figure S1. Chronic stress reduces the diversity of intestinal microorganism in the P10 group. Alpha diversity analysis of intestinal microorganism chronic stress. (a) Shannon curve, rarefaction curve, and rank abundance curve in the P10 group after chronic stress (n=3-5). (b) ACE index, Simpson index, Shannon index, and Chao1 index in the P10 group after chronic stress (n=3-5). (c) The relative abundance of Bacteroides, *Camobacteriaccae*, *Prevotellaceae*, *Ruminococcaceae*, *uncultured_bacterium_g_Staphylococcus*, and *Alistipes* in the P10 group after chronic stress (n=3-5).
